# A Bio-Inspired Bistable Piezoelectric Structure for Low-Frequency Energy Harvesting Applied to Reduce Stress Concentration

**DOI:** 10.3390/mi14050909

**Published:** 2023-04-23

**Authors:** Nan Wu, Jiyang Fu, Chao Xiong

**Affiliations:** Reserch Center for Wind Engineering and Engineering Vibration, Guangzhou University, Guangzhou 510006, China; wunan@gzhu.edu.cn (N.W.);

**Keywords:** low frequency, s-type, bistable, energy barrier

## Abstract

Inspired by the two typical movement stages in the wingbeat cycle of a seagull in flight, a bio-inspired bistable wing-flapping energy harvester is proposed in this paper to effectively convert low-frequency, low-amplitude and random vibrations into electricity. The movement process of this harvester is analyzed, and it is found that it can significantly alleviate the shortcomings of stress concentration in previous energy harvester structures. A power-generating beam composed of a 301 steel sheet and a PVDF (polyvinylidene difluoride) piezoelectric sheet with imposed limit constraints is then modeled, tested and evaluated. The energy harvesting performance of the model at low frequencies (1–20 Hz) is experimentally examined, where the maximum open-circuit output voltage of the model reaches 11,500 mV at 18 Hz. With a 47 kΩ external resistance of the circuit, the peak output power of the circuit reaches its maximum state of 0.734 mW (18 Hz). When a full bridge circuit is employed to convert AC to DC, the 470 μF capacitor connected to it reaches 3000 mV at peak voltage after 380 s of charging.

## 1. Introduction

The integration of self-powered sensors into the Internet of Things (IoT) environment can have a profound impact [1]. Wireless sensing nodes are generally powered by batteries, which have limited energy storage and require periodic replacement, limiting the use of wireless sensors. As electronic devices have become increasingly energy-efficient, it has become possible to convert energy from the environment into electricity in order to power low-power devices. Vibrational energy, which has a high energy density, is ubiquitous and easy to utilize, and is widely present in transportation, industrial equipment, and biological motion, among others. Vibration energy, being widely present, is a potential source of energy that could be harvested to power sensors [2,3,4]. The production of low-frequency energy harvesters is essential, as the vibration sources in ambient conditions, such as human walking, water flow, heartbeats, wind, or vehicle tires, tend to be in the range of 0–20 Hz [5,6]. This is of particular importance considering the development of wireless self-powered sensors for the IoT.

Linear resonators can be used to enhance the response of energy harvesters. Therefore, scholars have designed linear piezoelectric and electromagnetic energy-harvesting structures to improve the energy harvesting effect of mechanical equipment, wind, waves, and other stimuli [7,8,9]. Song et al. [10,11] proposed a piezoelectric energy harvester consisting of a vertical cylinder that generates vortex-induced vibration in the incoming flow and drives a piezoelectric beam to oscillate back and forth. Due to the parallel connection of the piezoelectric beam and the oscillator, a Type I structure is formed, effectively increasing the vibration response of the harvester. Sun et al. [12] demonstrated through experiments that flow velocity and load resistance play vital roles in power harvesting. In addition to fluid–structure interaction, the electromechanical coupling coefficient also significantly affects the system’s damping. Molino et al. [13] proposed a double-pendulum piezoelectric energy harvester, wherein the lower cylinder reduces the device’s natural frequency, making it more susceptible to vortex-induced resonance in low-speed water flow and able achieve large-scale oscillation. Test results showed that the energy harvesting efficiency was about 0.1%. Kim et al. [14] found that the impact of increasing or decreasing the oscillator’s mass on the harvester performance is much greater than changing the cantilever beam parameters, which reduces frequency and improves the energy harvesting efficiency.

However, linear energy collectors usually have a narrow bandwidth around the resonance frequency, resulting in a lower harvesting efficiency. In general, the stimulus frequency in the surrounding environment randomly changes within a certain bandwidth, making it impossible to effectively capture energy outside the resonance frequency [15]. In order to broaden the energy harvesting bandwidth and improve the energy harvesting efficiency, scholars have designed multiple cantilever beam arrays, L-shaped beams and multi-degree-of-freedom beams, and have proposed passive, semi-active, and adaptive methods [16,17,18]. The main principle is to increase multiple resonance frequency points in the system to broaden the resonance frequency band. However, these methods increase the mass and use space of the system, make the structural system too complex, and reduce the energy harvesting efficiency per unit mass and volume. Additionally, the problem of the narrow energy harvesting range under a single resonance frequency has not yet been resolved [19,20,21].

To enhance the efficiency of energy harvesting, amplitude/frequency amplification technology and nonlinear dynamics technology are two popular approaches. Amplitude/frequency amplification technology can improve the performance by increasing the amplitude and frequency of the energy harvest. Umeda et al. [22] presented a method of transforming the mechanical impact energy into electric energy via a piezoelectric transducer. Nonlinear harvesters with monostable [23,24,25], bistable [26,27], and multi-stable [28] behaviors have also been developed to modify the potential form of the harvesters by supplying pre-tightening forces or pre-deformation. For example, a clamped beam, designed by She et al. [29], can switch between monostable and bistable states according to the load applied to it. Leadenham et al. [30] and Yao et al. [31] adopted experimental [32] and theoretical [33] methods to analyze the dynamic characteristics and working effectiveness of a bistable vibration energy harvester, respectively. Kan et al. [34] proposed an improved piezoelectric wind-induced vibration energy harvester through the interplay between a cylindrical shell and diamond-shaped element. Wu et al. [20] put forward a novel bistable piezoelectric energy harvester that is susceptible to snap-through events so as to harvest wind energy from the environment. Additionally, Peng et al. [21] explored the impact of the frequency up-conversion effect on piezoelectric stack generators to yield high-performance energy harvests.

Moreover, bionic designs have been adopted to create piezoelectric energy harvesters [35]. Taking cues from the flight mechanism of dipterans, Zhou et al. [36] proposed a bionic dipteran energy harvester to convert low-frequency vibration energy. In addition, Fu et al. [37] designed a host–parasite vibration harvester by incorporating bistability and frequency up-conversion to capture random low-frequency vibrations. 

The present work proposes a novel bionic wing-flapping energy harvester for the effective conversion of low-amplitude, low-frequency random vibration into electricity. This paper is organized as follows. Section 2 explains the bionic design, mathematical modeling, and FEM optimization of the harvester. Section 3 describes the model’s characteristics in terms of stress concentration prevention. In Section 4, the experimental results of the harvester’s energy performance in various conditions are discussed. Lastly, Section 5 concludes this paper with some remarks.

## 2. Bio-Inspired Design and Mechanical Model

### 2.1. Bio-Inspired Design of the Wing-Flapping Energy Harvester

A good example of the effective external force obtained via low-frequency nonlinear motion in nature is the flight of seagulls [38]. The frequency of the fluttering flight of large birds such as seagulls is relatively low. During flight, because the wingspan determines the flutter frequency, the wingbeat frequency of seagulls is about 5 Hz, with the lowest being 1–2 Hz [39]. Per observation, during the wingbeat process of seagulls, not only do the wings beat up and down, but they also have a small amplitude of near-linear twist according to the various air resistances formed up and down, with the largest twist amplitude being at the tip of the wing and becoming lower closer to the body. This enables seagulls to obtain enough lift whilst maintaining a low wing-flapping frequency. 

**Figure 1:**
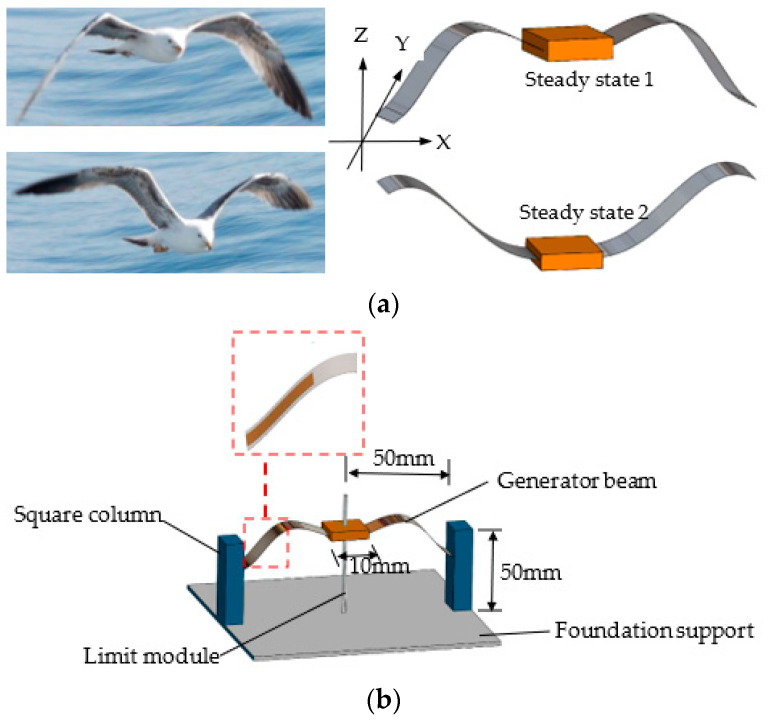
Schematic diagram of a bio-inspired bistable wing-flapping energy harvester: (**a**) two steady states of the generator beam of the harvester; (**b**) the harvester design in 3D.

A seagull in flight has two typical movement stages in the wingbeat cycle of its wings: lower and upper wing states (Figure 1a). Inspired by the wing beat of seagulls, the wing-flapping bionic energy harvester also has two steady states; when the harvester is excited by the environment, it can perform either small-amplitude motions within a single potential well (intra-well motion), or large-amplitude motions between the two potential wells (inter-well motion). The occurrence of inter-well motions requires breaking through potential energy barriers (snap-through), which constitutes a typical bistable structure.

By learning and mimicking the wingbeat cycle of a seagull in flight, which consists of two typical movement stages, a bio-inspired bistable wing-flapping energy harvester was proposed to effectively convert low-frequency, low-amplitude, random vibrations into electricity (as shown in Figure 1b). Piezoelectric polyvinylidene fluoride films (IPS-17020, Zhimei-Kang Co., Ltd, Shenzhen, China) were attached to the root of a seagull-shaped power-generating beam to convert mechanical energy into electrical energy. A suitable environmental stimulus triggers snap-through, causing the beam to change from one stable state to the other. This process creates a large amplitude of oscillation and a local high-frequency vibration, allowing the harvester to output greater electrical energy.

### 2.2. Mechanical Model

**Figure 2:**
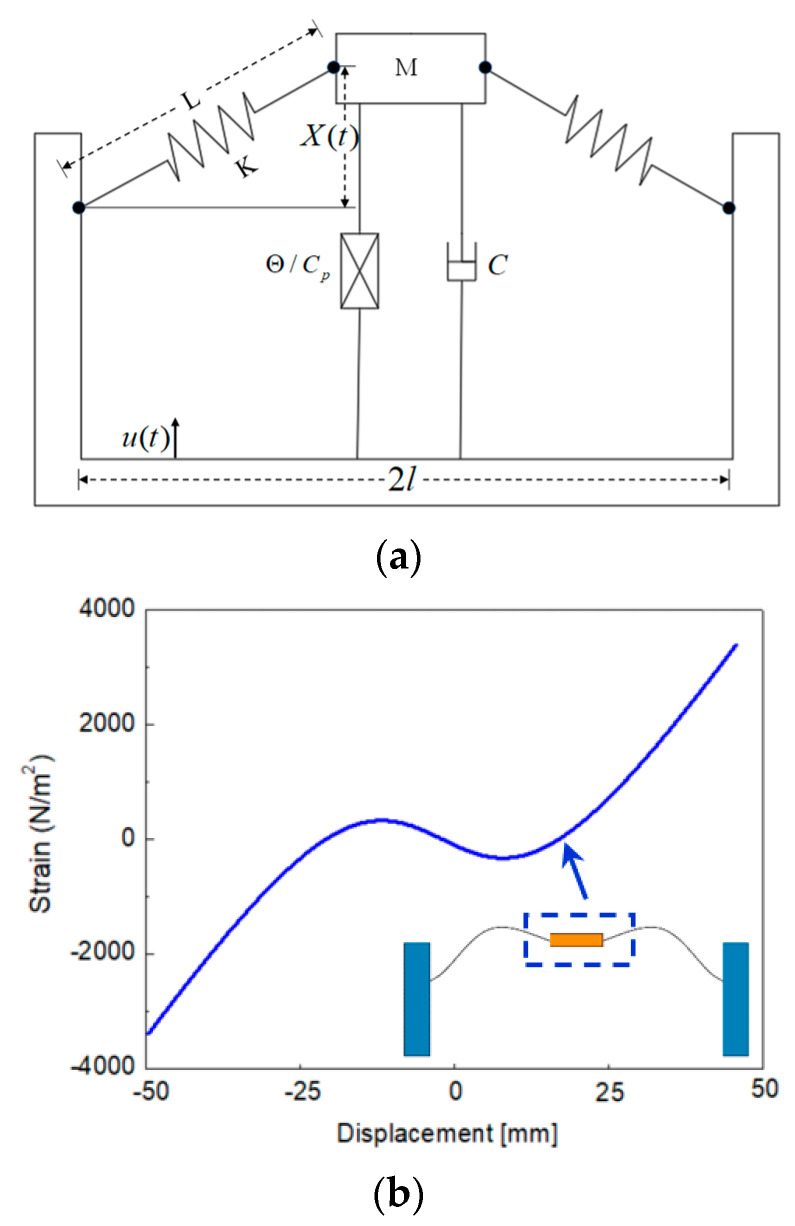
Mechanical model of the energy harvester: (**a**) piezoelectric coupled oscillator model; (**b**) the force curve of the mass block during one-time inter-well motion.

The abovementioned dynamic characteristics of the harvester may be simplified as a piezoelectric vibrator supported by an inclined spring, as shown in Figure 2a. Considering the influence of gravity, according to Newton’s second law and Kirchhoff’s law [31,32], the dynamic control equation of the piezoelectric spring mass system can be written as follows:

(1) $$\left\{\begin{array}{c} MX^{\prime \prime }+CX^{'}+2KX(\frac{L}{\sqrt{l^{2}+X^{2}}}-l)+\Theta V+Mg=MZ^{\prime \prime }\\ C_{P}V^{'}+\frac{V}{R}-\Theta X^{'}=0 \end{array}\right.$$

where *M* is the mass of the piezoelectric vibrator (the mass block sliding up and down along the limit rod in the middle of the power generation beam), *X* is the amplitude of the generator beam, *K* is the equivalent stiffness, *C* is the damping coefficient, *l* is the length of the generator beam, *g* is the gravitational acceleration constant, *μ(t)* is the displacement of the external vibration source as a function of time, *V* is the output voltage of the piezoelectric sheet, *Θ* is electromechanical coupling coefficient, and *Cp* is the equivalent capacitance.

The dimensionless parameter $c_{v}$, $x=L\bar{x},\;u=La,\;V=c_{v}v,\;\tau =\sqrt{2K/Mt}$, is introduced when the spring is compressed, α ≠ 0. The irrational item is Taylor expanded at $\bar{X}=0$, $\alpha \prime \prime =f\cos(w\tau ),\;f=\frac{A}{L},\;w=\Omega \sqrt{\frac{M}{2K}}$; thus, the above equation is rewritten as follows:

(2) $$\left\{\begin{array}{l} {\bar{x}}^{\prime \prime }+2\xi {\bar{x}}^{\prime }+(1-\frac{1}{\alpha })\bar{x}+\frac{{\bar{x}}^{3}}{2\alpha^{3}}+\rho +\theta \upsilon =f\cos(\omega \tau )\\ \upsilon^{\prime }+\lambda \upsilon -\beta {\bar{x}}^{\prime }=0 \end{array}\right.$$

Transforming $x^{\prime }=y$, Equation (2) can be transformed into an equation of state:

(3) $$\left\{\begin{array}{l} x^{\prime }=y\\ y^{\prime }=-2\xi y+\alpha_{1}x+\alpha_{2}x^{2}+\alpha_{3}x^{3}-\theta v+f\cos(\omega \tau )\\ \upsilon^{\prime }=-\lambda \upsilon +\beta y \end{array}\right.$$

Based on the above model, the stress state of the bistable generator beam of the energy harvester during a period of motion (inter well motion) was calculated, as shown in Figure 2b. As the generator beam of the energy harvester moved from the lower steady state to the upper steady state, the force on the mass block changed from −3460 N/m^2^ to 3460 N/m^2^. It is obvious from the figure that the force on the mass block changes when the snap-through occurs (the generator beam breaks through the potential energy barrier).

### 2.3. FEM Optimization Design

**Figure 3:**
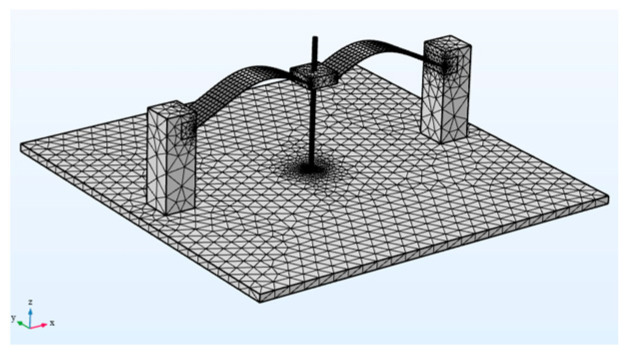
Finite element simulation model.

**Figure 4:**
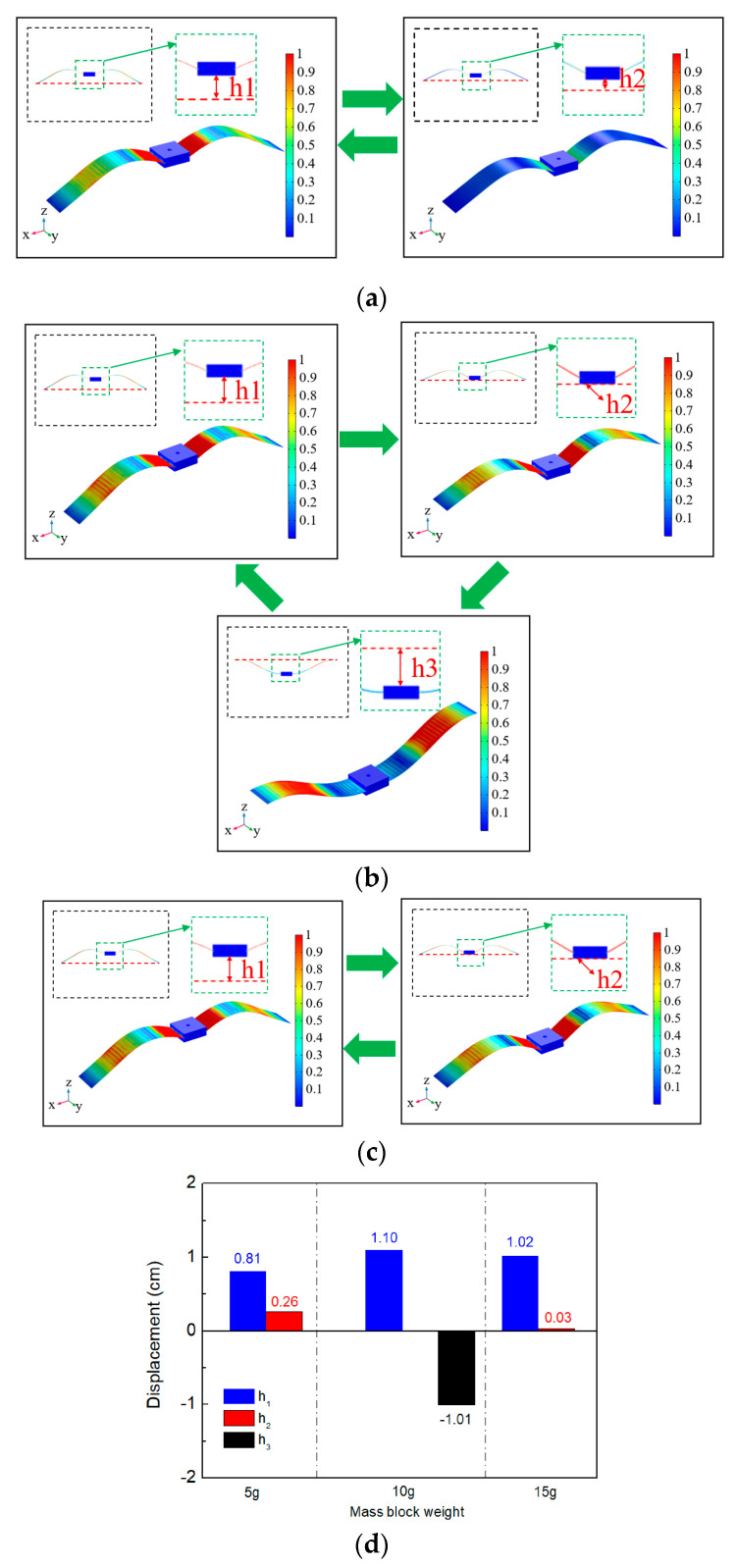
The displacement characteristic of generator beam with different mass weights: (**a**) 5 g; (**b**) 10 g; (**c**) 15 g; (**d**) displacement characteristic histogram.

To analyze and optimize the structure of the wing-flapping bionic energy harvester, we conducted finite element method (FEM) simulations using COMSOL Multiphysics 5.3 (Table 1 and Figure 3). Using built-in solvers of the frequency domain, the optimum mass parameters were determined by simulating and analyzing the movement process of the middle mass block of the generation beam at different weights (5 g, 10 g and 15 g). As shown in Figure 4, h_1_ represents the maximum distance between the middle mass block of the generation beam and the centerline (the connecting line between the two ends of the generation beam); h_2_ represents the shortest distance of the middle mass block of the generation beam above the centerline; and h_3_ represents the maximum distance of the middle mass block of the generation beam below the centerline.

Using software, fixed constraints were applied at both ends of the bistable wing-flapping power generation beam, and a mass block was added at the middle. A low-frequency vertical excitation (0–20Hz) was then applied to the beam using COMSOL 5.3. The motion states of the power generation beam were analyzed in different mass block weights (5 g, 10 g, and 15 g) in order to determine the optimal height and geometric shape of the potential well. To clearly demonstrate the motion states of the beam under different geometric sizes, the cloud diagram data was normalized; the legend values represent the relative motion states of the power generation beam at different extreme positions.

From Figure 4a, it can be seen that when the weight of the mass block was 5 g, only the in-well movement of the generation beam could occur, which could not break through the potential barrier. The mass block movement ranges was between h_1_ (0.81 cm) and h_2_ (0.26 cm) (Figure 4d). When the weight of the mass block was increased to 10 g (Figure 3b), the movement process of the power generation beam broke through the potential barrier and changed from the original in-well motion to the inter-well motion. The amplitude of the power generation beam was remarkably large. When snap-through occurred, it was accompanied by the local high-frequency vibration of the generation beam. Correspondingly, the mass block movement range was between h_1_ (1.10 cm) and h_3_ (−1.01 cm) (Figure 4d). Further, when the weight of the mass block was 15 g (Figure 3c), the beam could only move in the well, and the mass block motion ranged between h_1_ (1.02 cm) and h_2_ (0.03 cm) (see Figure 4d).

## 3. Relief of Stress Concentration

**Figure 5:**
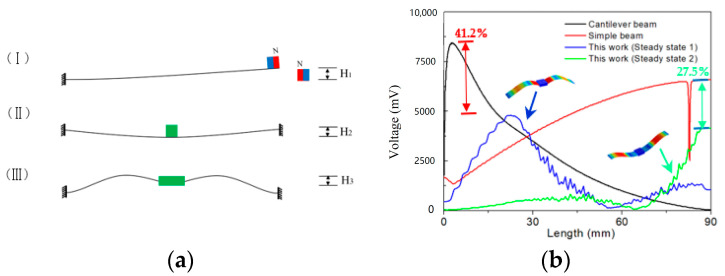
Stress analysis of power generation beam: (**a**) schematic diagram of a typical bistable power generation beam; (**b**) the stress distribution of the generator beam during movement (inter-well motion).

Currently, the generator design based on the bistable (multi-stable) principle mostly extends the power generation structure based on the cantilever beam (Figure 5a (I) or arch beam (Figure 5a (II). This structure has certain limitations in terms of fatigue life and spatial utilization. However, the root of the cantilever beam is prone to fatigue fracture after long-term large-amplitude vibration, and the root cause is the long-term stress concentration produced by this type of structure during the motion of the energy collector; similarly, the dynamic device of the arch beam also has stress concentration areas during the motion process, cannot fully utilize the material strength, and has premature fatigue failure problems. 

This work was based on the seagull-inspired design of the power generation beam, which can effectively improve the uniformity of stress during the motion of the power generation beam and alleviate stress concentration phenomena compared to the first two devices (Figure 5a (III)), thus effectively extending the material’s service life. The finite element method was used to compare the stress changes (same potential well height, i.e., H_1_ = H_2_ = H_3_; geometric dimension: 90 mm × 20 mm × 1 mm; end mass: 10 g) during the motion of the seagull-inspired beam proposed in this paper and the typical cantilever and arch beams (the two ends of the arch beam are non-rigidly connected). 

When the three types of power generation beams reached their respective resonance frequencies, the maximum stress curve of the beams during the motion could be obtained, as shown in Figure 5b. The cantilever beam and arch beam showed obvious stress concentration areas during the motion process; even if the beam was moved, the position of the stress concentration remained unchanged. In contrast, the peak stress position of the seagull-inspired beam proposed in this paper continuously moved with the variation in the motion process (from steady state 1 to steady state 2), and could fully utilize materials and avoid the stress concentration that causes the premature fatigue failure of materials. In addition, in terms of peak stress, the peak stress of the seagull-inspired beam was 41.2% lower than that of the cantilever beam and 27.5% lower than that of the arch beam, indicating the huge potential of the seagull-inspired beam to improve the material fatigue failure.

## 4. Experiments and Analysis

### 4.1. Prototype Fabrication and Experimental Setup

**Figure 6:**
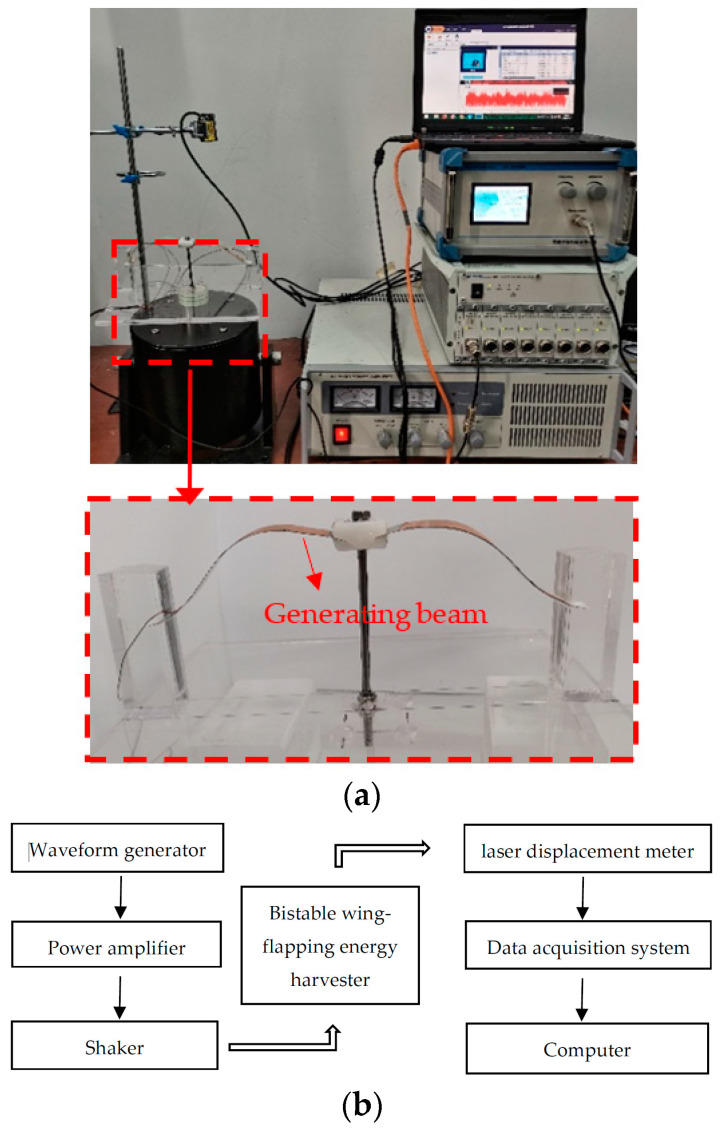
Experimental platform and testing: (**a**) experimental prototype and set-up; (**b**) test schematic diagram.

As shown in Figure 6, a prototype was made and tested under different vibration conditions to evaluate its energy harvesting performance. The electricity-generating part of the harvester was composed of two generator beams placed in between the embedded limit module, which were constructed using piezoelectric polyvinylidene fluoride films (IPS-17020, size: 13 mm × 25 mm; wideband 0.001 Hz–1000 MHz; high sensitivity; accuracy: 1.4 V/g~16 V/g; operating temperature range: 0~70 °C) adhered on the steel plate (material: 301; thickness 0.1 mm) [3]. The limit module was composed of 10 g of resin block made of poly methyl methacrylate (PMMA), which could move up and down along the steel bar fixed on the base. Furthermore, the two ends of the generator beam were connected to the base through two square column embedments. The power generation beam was embedded into the column body through a groove that was reserved on the side of the square column. Once the embedding was completed, the groove was injected with glue to completely fix the connection, which could be considered a rigid connection. The distance between the two square columns made the generator beam undergo bionic pre-deformation that was similar to the shape of a gull wing.

The environmental excitation of the prototype was provided by an SA-JZ020 shaker (Wuxi Shiao Technology Co., Ltd., Shenzhen, China). The bottom plate of the model was fixed to the top rod of the exciter with screws. An SA-SG030 signal generator, which generated an input signal, was amplified by an SA-PA080 amplifier (Wuxi Shiao Co., Ltd., Shenzhen, China) and fed to the shaker [3]. The parameters of the equipment were as follows: maximum exciting force of 200 N, maximum acceleration of 30 g, force constant of 14.3 N/A, maximum amplitude of ±10 mm, and frequency range of 0–2000 Hz [3]. The displacement of the generator beam was measured using an HG-C1200 laser displacement meter (the measurement center distance and measurement range of the laser displacement sensor were 30 ± 5 mm, and the repetition accuracy was 10 μm. The beam diameter was 50 μm), which was fixed above the test bench.

### 4.2. The Snap-Through Phenomenon

**Figure 7:**
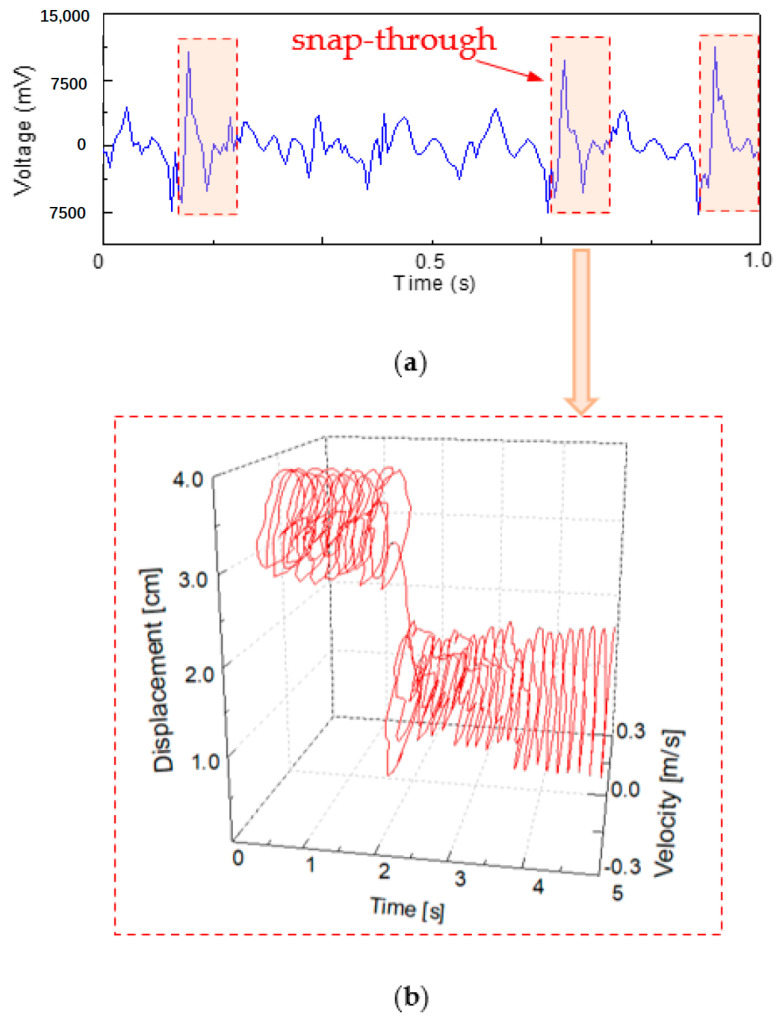
The snap-through phenomenon: (**a**) the voltage signal of generator beam during movement; (**b**) the displacement and velocity of the generator beam in the snap-through moment.

The flapping wing bionics energy harvester is a bistable energy capture structure. As shown in Figure 7a and steady state 1 and steady state 2, there are two stable states of the energy harvester. When the external excitation is insufficient to make the power generation beam break through the potential energy barrier, the beam can only move in the well, that is, vibration occurs appears as shown in Figure 4a or Figure 4c. 

When the external excitation conditions change, a sudden snap-through phenomenon occurs in the power generation beam, which changes from the movement in the well to the movement between the wells (i.e., vibration occurs between the states shown in Figure 4b. The voltage signal output by the power generation beam is shown in Figure 7a. The peak voltage range jumped from 3500~3900 mV to 11,000~11,500 mV due to the influence of the sudden bounce snap-through phenomenon). In the case of snap-through, the system dynamics are illustrated in Figure 7b. In a continuous time process, the motion track of the energy harvester generator beam suddenly jumps from a track center at the 3.5 cm displacement coordinate to a track center at the 1.5 cm displacement coordinate. The instantaneous migration of the whole motion track is realized.

### 4.3. Voltage Frequency Response Analysis

**Figure 8:**
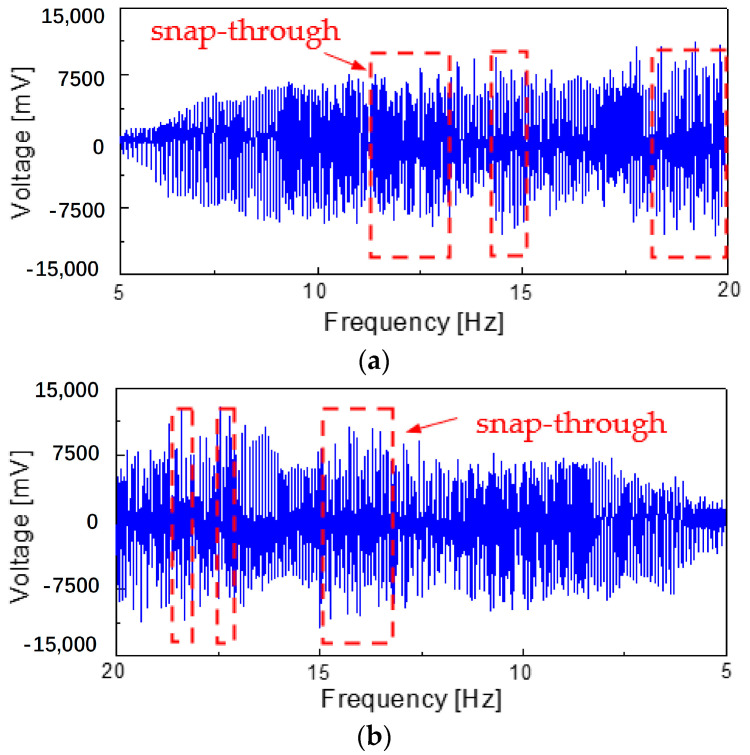
The voltage signal output from generator beam under sweep excitation (5–20 Hz): (**a**) forward sweep; (**b**) backward sweep.

In order to comprehensively evaluate the working performance of the energy harvester at the low-frequency state, the low-frequency excitation of 5~20 Hz was applied to the energy harvester model, as shown in Figure 8. With the increase in the excitation frequency, the amplitude of the generator beam moving in the well increased, and the peak value of the output voltage also increased (Figure 8a). When the excitation frequency increased to 8 Hz, the output voltage of the generator beam moving in the well reached the maximum value (output voltage is −7800~7400 mV). Furthermore, with a further increase in the excitation frequency, the motion state of the power generation beam tended to be irregular, but it was always unable to break through the potential energy barrier and was trapped in the well vibration. However, when the frequency reached 11 Hz, this state was completely changed. At this time, the power generation beam broke through the potential energy barrier and a snap-through occurred (11~13 Hz); this changed from the in-well motion to the inter-well motion. When the excitation frequency was between 13 and 14.5 Hz, the motion state of the generator beam changed to the in-well motion again. However, with the increase in the excitation frequency to 14.5~15 Hz and 17.5~20 Hz, the generator beam continued to snap-through and changed to cross-well motion in this frequency range.

When the external excitation load was applied to the model from high to low (20~5 Hz), the power generation beam showed a change in the law of motion state that was roughly opposite to the forward sweep excitation (5~20 Hz). However, the difference was that the power generation beam had a snap-through in the range of 18.5 Hz, 17 Hz and 13~15 Hz, respectively, from in-well motion to inter-well motion

**Figure 9:**
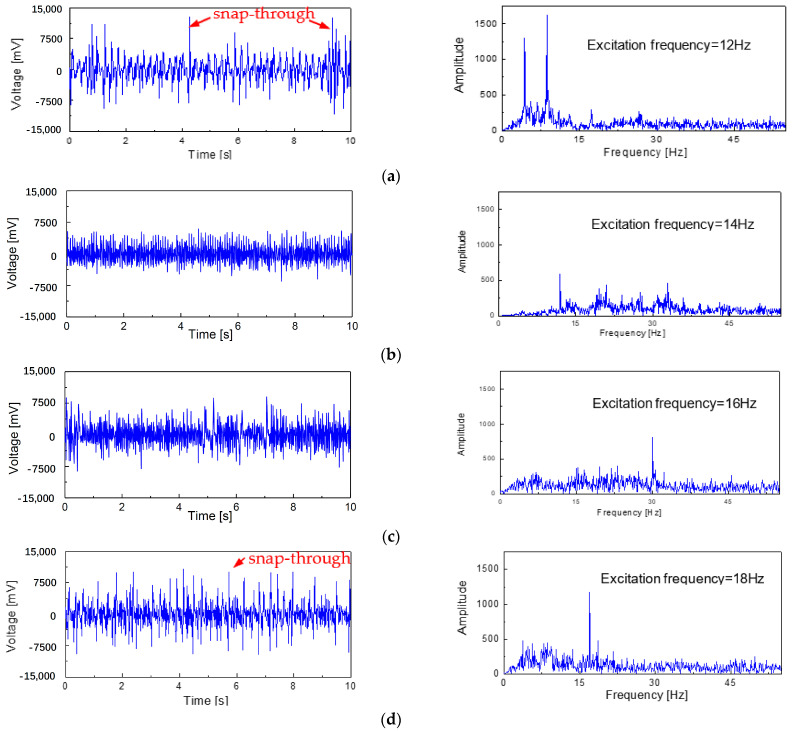

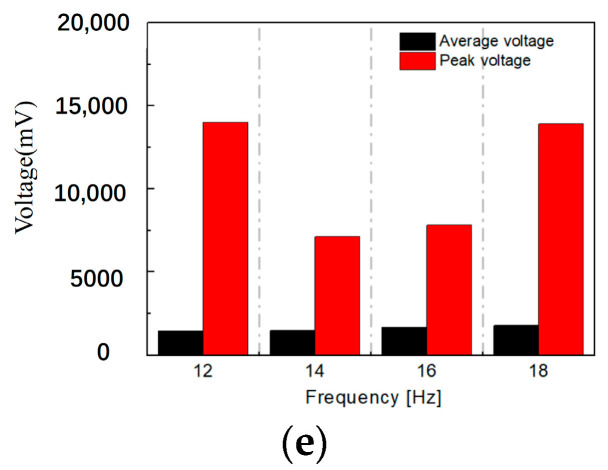
The open-circuit voltage waveforms and the FFT at different excitation frequencies: (**a**) 12 Hz, (**b**) 14 Hz, (**c**) 16 Hz, and (**d**) 18 Hz; (**e**) the voltage signal histogram.

The typical waveforms of the output voltage varying with time are shown in Figure 9. When the external excitation frequency was 12 Hz, the changes in the output voltage signal of the generator beam with time and the fast Fourier transform (FFT) could be discerned, as shown in Figure 9a. It was found that there was a sharp jump in the output voltage signal that was caused by the snap-through phenomenon occurring on the generator beam; however, this is rare and irregular. As the excitation frequency increased to 14 Hz, it can be seen from Figure 9b that the density of the output peak voltage signal increased, but no snap-through phenomenon occurred. When the excitation frequency was further increased to 16 Hz (Figure 9c), it can be seen that part of the voltage signal reached the critical state at which the generator beam experiences a snap-through phenomenon. Until the external excitation frequency of 18 Hz was applied to the model (Figure 9d), the sharp rise in the output voltage signal caused by the snap-through phenomenon frequently occurred. The signal output characteristics of the generator beam with these typical excitation frequencies show the same change rule as the sweep excitation.

### 4.4. Output Performance of the Designed Harvester

**Figure 10:**
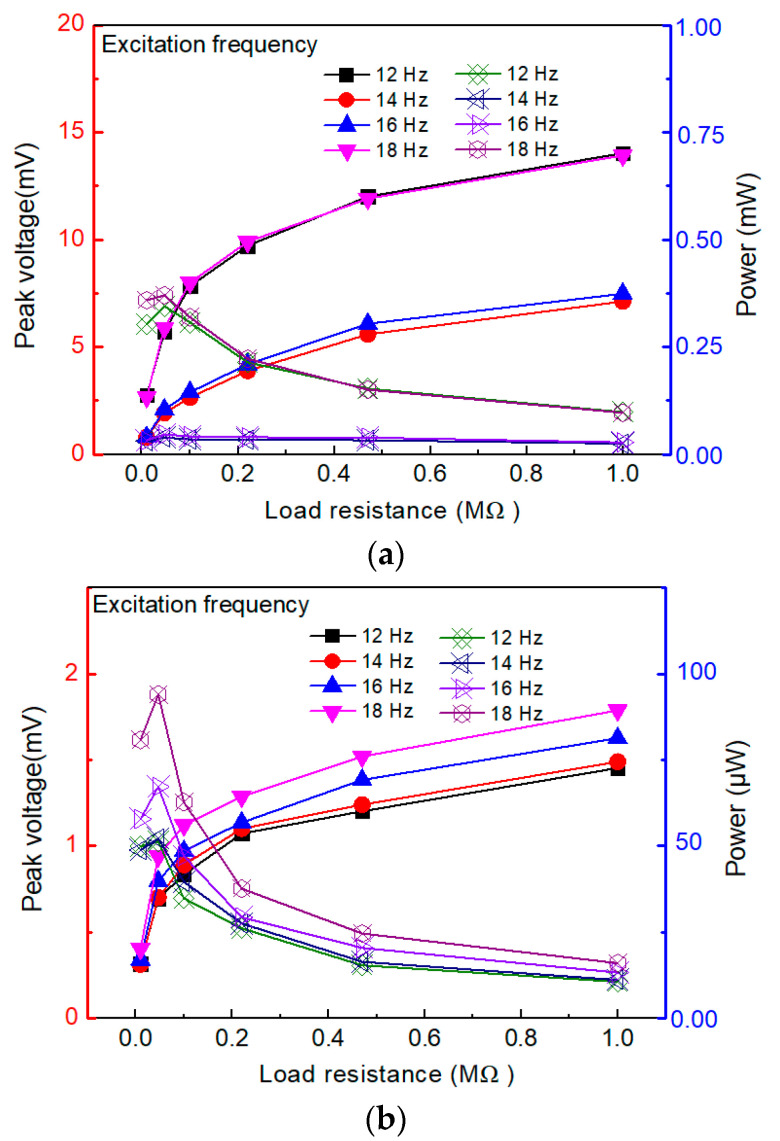
The output performance of the harvester at different circuit load conditions: (**a**) peak value of voltage and power; (**b**) mean value of voltage and power.

The performance of the designed energy-harvesting device was evaluated based on the average output power of the harvester under different testing conditions. The curves of the output voltage and power of the harvester under different circuit loading conditions are shown in Figure 10 (the curves on which the solid points are located correspond to the voltage signal (on the left *y*-axis), and the curves on which the hollow points are located correspond to the average output power (on the right *y*-axis)).

The results of this study showed that the external resistance has an important impact on the output voltage and power of the energy harvester. As shown in Figure 10a, the peak output voltage increased as the load resistance increased, while the peak output power displayed an increasing trend and then a decreasing trend. When the external resistance of the circuit was 47 kΩ, the peak output power reached its maximum value of 0.734 mW (18 Hz). Thus, the average output voltage and power exhibited a similar behavior to that of the variation in the load resistance, which is due to the close relation between the optimal load resistance of the piezoelectric energy generator and the resonance frequency of the structure, as well as the capacitance of the piezoelectric element. The average power *P_avg_* is calculated as $P_{avg}=\frac{V^{2}{}_{rms}}{R}$, where $V_{rms}=\sqrt{\frac{1}{T_{2}-T_{1}}\int_{T_{1}}^{T_{2}}V^{2}dt}$ (*V*: the voltage, *R*: the resistance) denotes the root mean square (*RMS*) voltage (*V*) [3,9]. The results showed that the output power firstly increased, then decreased, and reached its maximum when the external resistance was 47 KΩ.

**Figure 11:**
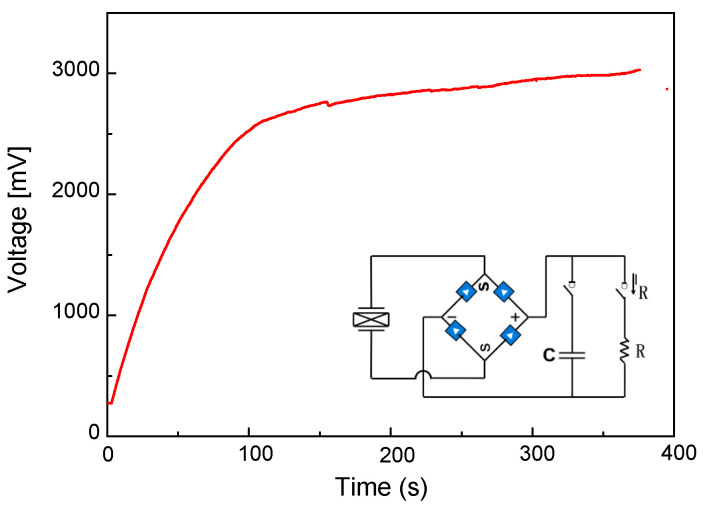
The output performance through a rectifier circuit.

To verify the energy-harvesting capabilities of the model, a 470 μF capacitor was used to gather the electrical energy when the excitation frequency was 18 Hz. A full bridge circuit was used to change the alternating current (AC) to a direct current (DC), as shown in Figure 11. The charging continued for about 380 s and the maximum voltage of the capacitor reached 3000 mV. Furthermore, it was found that in the first 100 s, the charging speed increased rapidly with the increase in time, and the charging speed slowed down significantly compared with the previous period of time in 100~380 s. 

This work was based on the bionic principle and used a bistable nonlinear mechanism to design an energy harvester that comprised inexpensive and easily available manufacturing materials; thus, it had the advantages of a low manufacturing cost and good application prospects. In addition, a performance comparison with several typical energy harvesters was performed reported (Table 2). Through comparison, it was found that the energy trap proposed in this article has the characteristics of a low resonant frequency (18 Hz), moderate internal resistance, and a high energy capture efficiency (0.734 mW).

## 5. Conclusions

In conclusion, a bio-inspired bistable energy harvester has been designed, prototyped and tested to assess its ability to energy from broadband vibrations. The following are the most crucial outcomes of this study:

1. When the mass of the middle beam of the energy harvester is 10 g, snap-through is observed when the motion of the power generator (the movement range in the mass block is between h_1_ (1.10 cm) and h_3_ (−1.01 cm)) changes from the original in-well motion to the inter-well motion, exceeding the potential barrier (snap-through).
2. The peak stress position of the seagull-inspired beam proposed in this paper can fully utilize materials and the avoid stress concentration that causes the premature fatigue failure of materials. In addition, in terms of peak stress, the peak stress of the seagull-inspired beam is 41.2% lower than that of the cantilever beam and 27.5% lower than that of the arch beam, indicating the huge potential of the seagull-inspired beam to improve material fatigue failure.
3. When the external resistance of the circuit is set to 47 kΩ, the peak output power of the circuit achieves the maximum state of 0.734 mW (18 Hz). As a consequence, when the full bridge circuit that converts AC to DC is sent to a 470 μF capacitor, the maximum voltage of the capacitor can reach 3000 mV after 380 s of charging.

## Figures and Tables

**Table 1:** Simulation parameters of bionic gull wing harvester with single degree of freedom.

Material	Property
Stainless steel sheet	Density ρ = 7850 kg/m^3^; Young’s Modulus E = 190 Gpa; Poisson’s ratio ν = 0.30;Width b1 = 20 mm;Thickness h1 = 0.1 mm
PVDF	Density ρ = 1780 kg/m^3^; Relative permittivity {epsilonrS11, epsilonrS22, epsilonrS33} {7.4 9.3 7.6}; Width b2 = 20 mm;Thickness h2 = 0.1 mm

**Table 2:** Performance comparison of various energy harvesters in previous reports.

References	Operation Mechanism	Material	Central Frequency (Hz)	Power (μW)	Impedance (Ω)
Zhang et al. [40]	Piezoelectric	PZT-5H	21, 31	700, 530	60 k, 29 k
Cai et al. [41]	Piezoelectric	PVDF	24	129.4	600 k
Fu et al. [42]	Electromagnetic	Magnet, coil	1~3	8	N/A
Tian et al. [43]	Electromagnetic	Magnet, coil	12.8	133	N/A
This work	Bi-stability	Steel and PVDF	18	734	47 k

## Data Availability

The introduction data supporting this manuscript are from previously reported studies and datasets, which have been cited. The processed data are available from the corresponding author upon request. The test raw data used to support the findings of this study are available from the corresponding author upon request.

## References

[1] Rui X., Li Y., Liu Y., Zheng X., Zeng Z. (2018). Experimental Study and Parameter Optimization of a Magnetic Coupled Piezoelectric Energy Harvester. Appl. Sci..

[2] Ambrożkiewicz B., Litak G., Wolszczak P. (2020). Modelling of Electromagnetic Energy Harvester with Rotational Pendulum Using Mechanical Vibrations to Scavenge Electrical Energy. Appl. Sci..

[3] Wu N., He Y., Fu J. (2021). Bistable energy harvester using easy snap-through performance to increase output power. Energy.

[4] Speciale A., Ardito R., Baù M., Ferrari M., Ferrari V., Frangi A.A. (2020). Snap-Through Buckling Mechanism for Frequency-up Conversion in Piezoelectric Energy Harvesting. Appl. Sci..

[5] Liu F.R., Zhang W.M., Peng Z.K., Meng G. (2019). Fork-shaped bluff body for enhancing the performance of galloping-based wind energy harvester. Energy.

[6] Liu S., Wang W. (2021). Response Analysis of Asymmetric Monostable Harvesters Driven by Color Noise and Band-Limited Noise. Appl. Sci..

[7] Zhang B., Mao Z., Song B., Ding W., Tian W. (2018). Numerical investigation on effect of damping-ratio and mass-ratio on energy harnessing of a square cylinder in FIM. Energy.

[8] Usman M., Hanif A., Kim I.H., Jung H.J. (2018). Experimental validation of a novel piezoelectric energy harvesting system employing wake galloping phenomenon for a broad wind spectrum. Energy.

[9] Zhang L.B., Dai H.L., Abdelkefi A., Wang L. (2019). Experimental investigation of aerodynamic energy harvester with different interference cylinder cross-sections. Energy.

[10] Song R., Dan X., Li J., Xie T. (2016). Modeling and Experimental Study of Piezoelectric Energy Harvesterunder Vortex-Induced Vibration. J. Vib. Control.

[11] Song R., Dan X., Fan M., Xie T. (2017). Simulations and experiments on a hydrodynamic compound pendulum piezoelectricenergy harvester accompanied with vortex-induced vibration. J. Vib. Control.

[12] Sun W., Tan T., Yan Z., Zhao D., Luo X., Huang W. (2018). Energy Harvesting from Water Flow in Open Channel with Macro Fiber Composite. AIP Adv..

[13] Molino-Minero-Re E., Carbonell-Ventura M., Fisac-Fuentes C., Manuel-Lazaro A., Toma D.M. (2012). Piezoelectric Energy Harvesting from Induced Vortex in Water Flow. Proceedings of the 2012 IEEE International Instrumentation and Measurement Technology Conference Proceedings.

[14] Kim M., Hoegen M., Dugundji J., Wardle B.L. (2010). Modeling and Experimental Verification of Proof Mass Effects on Vibration Energy Harvester Performance. Smart Mater. Struct..

[15] Yildirim T., Ghayesh M.H., Li W., Alici G. (2017). A Review on Performance Enhancement Techniques for Ambient Vibration Energy Harvesters. Renew. Sustain. Energy Rev..

[16] Li P., Liu Y., Wang Y., Luo C., Li G., Hu J., Liu W., Zhang W. (2015). Low-Frequency and Wideband Vibration Energy Harvester with Flexible Frame and Interdigital Structure. AIP Adv..

[17] Liu D., Al-Haik M., Zakaria M., Hajj M.R. (2018). Piezoelectric Energy Harvesting Using L-Shaped Structures. J. Intell. Mater. Syst. Struct..

[18] Zhou S., Hobeck J.D., Cao J., Inman D.J. (2017). Analytical and Experimental Investigation of Flexible Longitudinal Zigzag Structures for Enhanced Multi-Directional Energy Harvesting. Smart Mater. Struct..

[19] Zhou S., Cao J., Inman D.J., Liu S., Wang W., Lin J. (2015). Impact-Induced High-Energy Orbits of Nonlinear Energy Harvesters. Appl. Phys. Lett..

[20] Wu N., He Y., Fu J., Peng L. (2021). Performance of a bistable flow-energy harvester based on vortex-induced vibration. J. Wind Eng. Ind. Aerodyn..

[21] Yan P., Zhibing X., Min W., Zhongjie L., Jinlin P., Jun L. (2021). Investigation of frequency-up conversion effect on the performance improvement of stack-based piezoelectric generators. Renew. Energy.

[22] Umeda M., Nakamura K., Ueha S. (1996). Analysis of the Transformation of Mechanical Impact Energy to Electric Energy Using Piezoelectric Vibrator. Jpn. J. Appl. Phys..

[23] Harne R.L., Wang K.W. (2013). A review of the recent research on vibration energy harvesting via bistable systems. Smart Mater. Struct..

[24] Pellegrini S.P., Tolou N., Schenk M., Herder J.L. (2013). Bistable vibration energy harvesters: A review. J. Intell. Mater. Syst. Struct..

[25] Andò B., Baglio S., Bulsara A.R., Marletta V. (2014). A bistable buckled beam based approach for vibrational energy harvesting. Sens. Actuators A-Phys..

[26] Harne R.L., Thota M., Wang K.W. (2013). Bistable energy harvesting enhancement with an auxiliary linear oscillator. Smart Mater. Struct..

[27] Qian F., Zhou S., Zuo L. (2020). Approximate solutions and their stability of a broadband piezoelectric energy harvester with a tunable potential function. Commun. Nonlinear. Sci..

[28] Tran N., Ghayesh M.H., Arjomandi M. (2018). Ambient vibration energy harvesters: A review on nonlinear techniques for performance enhancement. Int. J. Eng. Sci..

[29] She G.L., Yuan F.G., Ren Y.R., Xiao W.S. (2017). On buckling and postbuckling behavior of nanotubes. Int. J. Eng. Sci..

[30] Leadenham S., Erturk A. (2014). M-shaped asymmetric nonlinear oscillator for broadband vibration energy harvesting: Harmonic balance analysis and experimental validation. J. Sound. Vib..

[31] Yao M.H., Li Y.B., Zhang W. (2015). The steady-state response analysis of bistable piezoelectric converter. Appl. Mech. Mater..

[32] Cha Y., Chae W., Kim H., Walcott H., Peterson S.D., Porfiri M. (2016). Energy harvesting from a piezoelectric biomimetic fish tail. Renew. Energy.

[33] Qian F., Hajj M.R., Zuo L. (2020). Bio-inspired bi-stable piezoelectric harvester for broadband vibration energy harvesting. Energy Convers. Manag..

[34] Junwu K., Weilin L., Jin W., Shuyun W., Mengjia Y., Yonghua J. (2021). Enhanced piezoelectric wind-induced vibration energy 356 harvester via the interplay between cylindrical shell and diamond-shaped baffle. Nano Energy.

[35] Wang W., Wang X., He X., Wang M., Shu H., Xue K. (2019). Comparisons of bioinspired piezoelectric wind energy harvesters with different layout of stiffeners based on leaf venation prototypes. Sens. Actuators A-Phys..

[36] Zhou J., Zhao X., Wang K., Chang Y., Wen G. (2021). Bio-inspired bistable piezoelectric vibration energy harvester: Design and experimental investigation. Energy.

[37] Fu H., Zahra S.K., Ferri A. (2019). A bio-inspired host-parasite structure for broadband vibration energy harvesting from low-frequency random sources. Appl. Phys. Lett..

[38] Yang B., Yi Z., Tang G., Liu J. (2019). A gullwing-structured piezoelectric rotational energy harvester for low frequency energy scavenging. Appl. Phys. Lett..

[39] Li J.H., Fu G.Q., Wu B. (2020). Design and Simulation Analysis of Two Stage Bionic Flapping Wing Mechanism. Mach. Tool Hydraul..

[40] Zhang Z., Xiang H., Shi Z., Zhan J. (2018). Experimental Investigation on.Piezoelectric Energy Harvesting from Vehicle-Bridge Coupling Vibration. Energy Convers. Manag..

[41] Cai Y., Fu J., Wu N., Xiong C., Liu A., He Y. (2022). A High-Efficiency Curved Panel Energy Harvester Featured by Reduced Stress Concentration. Energy Convers. Manag..

[42] Fu H., Jiang J., Hu S., Rao J., Theodossiades S. (2023). A Multi-Stable Ultra-Low Frequency Energy Harvester Using a Nonlinear Pendulum and Piezoelectric Transduction for Self-Powered Sensing. Mech. Syst. Signal Process..

[43] Tian L., Shen H., Yang Q., Song R., Bian Y. (2023). A Novel Outer-Inner Magnetic Two Degree-of-Freedom Piezoelectric Energy Harvester. Energy Convers. Manag..

